# The Protein Network in Subcutaneous Fat Biopsies from Patients with AL Amyloidosis: More Than Diagnosis?

**DOI:** 10.3390/cells12050699

**Published:** 2023-02-22

**Authors:** Dario Di Silvestre, Francesca Brambilla, Francesca Lavatelli, Maila Chirivì, Diana Canetti, Claudia Bearzi, Roberto Rizzi, Johan Bijzet, Bouke P. C. Hazenberg, Vittorio Bellotti, Julian D. Gillmore, Pierluigi Mauri

**Affiliations:** 1 Institute for Biomedical Technologies (ITB), Biomedical Sciences, National Research Council (CNR), 20054 Segrate, Italy; 2 Department of Molecular Medicine, University of Pavia, Via Forlanini 6, 27100 Pavia, Italy; 3 Fondazione IRCCS Policlinico San Matteo, Viale Golgi 19, 27100 Pavia, Italy; 4 UOC Neurology, Fondazione Ca’Granda, Ospedale Maggiore Policlinico, Via F. Sforza, 28, 20122 Milan, Italy; 5 Department of Molecular Medicine, Sapienza University, Viale Regina Elena, 324, 00161 Rome, Italy; 6 Centre for Amyloidosis, Division of Medicine, University College London, London NW3 2PF, UK; 7 Fondazione Istituto Nazionale di Genetica Molecolare, Via F. Sforza 35, 20122 Milan, Italy; 8 Department of Medical Surgical Science and Biotecnologies, Sapienza University, 04100 Latina, Italy; 9 Amyloidosis Center of Expertise, University Medical Center Groningen, University of Groningen, 9713 GZ Groningen, The Netherlands; 10 Department of Rheumatology & Clinical Immunology, University Medical Center Groningen, University of Groningen, 9713 GZ Groningen, The Netherlands; 11 Scientific Direction, Fondazione IRCSS Policlinico San Matteo, 27100 Pavia, Italy

**Keywords:** amyloidosis, proteomics, systems biology, networks, PPI, co-expression

## Abstract

AL amyloidosis is caused by the misfolding of immunoglobulin light chains leading to an impaired function of tissues and organs in which they accumulate. Due to the paucity of -omics profiles from undissected samples, few studies have addressed amyloid-related damage system wide. To fill this gap, we evaluated proteome changes in the abdominal subcutaneous adipose tissue of patients affected by the AL isotypes *κ* and λ. Through our retrospective analysis based on graph theory, we have herein deduced new insights representing a step forward from the pioneering proteomic investigations previously published by our group. ECM/cytoskeleton, oxidative stress and proteostasis were confirmed as leading processes. In this scenario, some proteins, including glutathione peroxidase 1 (GPX1), tubulins and the TRiC complex, were classified as biologically and topologically relevant. These and other results overlap with those already reported for other amyloidoses, supporting the hypothesis that amyloidogenic proteins could induce similar mechanisms independently of the main fibril precursor and of the target tissues/organs. Of course, further studies based on larger patient cohorts and different tissues/organs will be essential, which would be a key point that would allow for a more robust selection of the main molecular players and a more accurate correlation with clinical aspects.

## 1. Introduction

Amyloidosis is characterized by the misfolding of circulating proteins, which are progressively deposited at the extracellular level as insoluble amyloid aggregates. The relationship between the site of production of the fibril precursor protein and its deposition allows for the distinction between localized and systemic amyloidosis, although some amyloidoses show both systemic and local features. Their current classification is based on the amyloid protein type, and, to date, 42 human amyloid fibril proteins have been identified in humans [1]. This knowledge is improving its diagnosis and, although systemic amyloidosis is classified as rare diseases, the increasing incidence in the elderly population could soon make it a new global health socio-economic problem [2].

The deposition of amyloid aggregates can occur in several organs, including the heart, which can be severely affected in structure and function, becoming the major survival determinant [3]. The process of amyloid formation and organ targeting and damage is multifaceted and still largely unknown. This makes understanding the pathogenic cascade complex and its control a challenge [4]. Due to the progressive nature of the disease, early diagnosis is therefore vital, and many studies have been made toward this aim [5]. For instance, patients with advanced heart damage have a significantly poorer outcome and more limited access to the most aggressive, yet most efficacious treatments [6].

To date, the diagnosis and typing of the systemic amyloidosis are performed, respectively, by Congo red staining and the mass spectrometry (MS)-based proteomics analysis of tissue biopsies, mainly subcutaneous fat aspirates [7]. No specific biomarkers of organ damage to specifically differentiate the distinct amyloidoses forms exist. However, several studies have been recently published, mainly for cardiac amyloidosis, focusing on classifying amyloid types based on circulating microRNAs [8,9,10], protein markers [11,12,13] and oligomers [14].

As for amyloidosis typing, amyloid deposits are often laser microdissected from biopsies prior to MS analysis [15]. If, on one hand, this sampling allows for the identification of non-fibrillar proteins enriched in the amyloid deposit, on the other hand, it is not sufficiently representative of molecular and histological alterations induced in tissue resident cells by the deposition of extracellular amyloid aggregates. Thus, although microdissection aims to improve diagnosis, it has led to a poor collection of -omics profiles at global organ and tissue level. Consequently, the integration of comprehensive multi-omics analyses to dissect pathological mechanisms in systemic amyloidosis is limited to few reports [16]. Most studies rely on proteomics data [17,18,19], while some of them have recently focused on RNAseq analysis and differentially expressed genes (DEGs), both in cultured cardiomyocytes exposed to amyloidogenic light chains [20] and in plasma cells from immunoglobulin light chain (AL) amyloidosis patients and normal controls [21].

Omics technologies and network analysis give the opportunity to investigate human diseases through data-derived system biology approaches based on graph theory [22]. When applied to complex tissues, these approaches provide the chance to evaluate, at a global level, the pathophysiological processes underlying the investigated phenotypes [23]. In the context of protein misfolding diseases, fruitful application examples are available for neurodegenerative diseases, such as Alzheimer’s [24,25,26,27] and Parkinson’s diseases [28,29,30]. Conversely, far fewer similar studies have been conducted analyzing biopsies from patients affected by systemic amyloidosis [7,18,19,21].

To fill this gap, in this perspective study, we investigated the proteome changes in abdominal subcutaneous adipose tissue of patients affected by AL amyloidosis at a system level. Along with transthyretin (TTR), myocardial infiltration by immunoglobulin light chains is the major cause of cardiac amyloidosis [31]. In addition to helping to shed light on pathogenic mechanisms subtending AL subtypes, we provided a pipeline for evaluating how protein fibril deposition might affect the physiological protein relationships in a tissue. To reach this goal, we exploited the correlation between a protein network’s structure and the functions that it supports [32]. Therefore, the knowledge previously acquired through protein profiling and quantitation was here integrated with the new insights inferred by modeling the proteome as protein–protein interaction (PPI) and co-expression networks. In particular, these models were combined and analyzed at a topological and functional level [22]. AL amyloidosis patients were further split into two groups based on amyloid typing as AL κ or λ type. This classification aimed to verify the correlation between different AL subtypes and the pathogenic mechanisms that they could trigger.

## 2. Materials and Methods

### 2.1. Collected Samples, Proteomic Analysis and Preliminary Functional Analysis

Abdominal subcutaneous adipose tissue protein profiles previously collected and analyzed were re-processed and used as reference dataset [7,17,18,33]. Specifically, *n* = 14 control subjects, *n* = 15 patients affected by ALκ amyloidosis and *n* = 15 patients affected by ALλ amyloidosis were considered to create a larger cohort of profiles never evaluated all together in a single study. Major details on samples origin, MS analytical parameters and raw mass spectra processing are reported in Supplementary Material.

A preliminary functional evaluation of the characterized protein profiles was performed by functional annotation tool contained in STRING database [34]. For each subject, enriched Reactome pathways were retained (FDR ≤ 0.05) [35]. They were compared by linear discriminant analysis (LDA) and those with F ratio ≥5 and *p*-value ≤ 0.01 were selected as differentially enriched among C, ALκ and ALλ groups.

### 2.2. Label-Free Quantitative Analysis

The characterized protein profiles were semi-quantitatively compared by a label-free approach, as previously reported [36]. To minimize the batch effect of samples collected at different times and analyzed with different instruments and methods, the spectral count (SpC) values were normalized using a total signal normalization method [37]. Data matrix dimensionality (44 subjects and 4319 proteins) was reduced by LDA and proteins with F ratio ≥ 3 and *p*-value ≤ 0.05 were selected as differentially abundant (DAPs); of note, to ensure a robust extraction of differentially abundant proteins (DAPs), only those identified in at least 50% of subjects were retained. Selected DAPs were further processed by principal component analysis (PCA) and Spearman’s correlation. Pairwise comparisons were finally evaluated by DAve index (see Supplementary Material). All data processing were performed using JMP 15.2 SAS software.

### 2.3. Reconstruction and Analysis of PPI and Co-Expression Network Models

A PPI network model per group (C, ALκ and ALλ) was reconstructed by considering proteins identified in more than 50% of subjects (per group). The network models were reconstructed by STRING Cytoscape’s APP [34] and only protein–protein interactions annotated with “databases” and/or “experiments” with a score ≥0.3 and ≥0.15 were retained. Similarly, a PPI network model was reconstructed starting from DAPs selected by LDA (*n* = 132, *p* ≤ 0.05). The proteins were grouped in PPI functional modules by the support of STRING Cytoscape’s APP and BINGO 2.44 [38]; *Homo sapiens* organism, hypergeometric test and Benjamini–Hochberg FDR correction (≤0.01) were set.

A protein co-expression network model per group was reconstructed processing the characterized protein profiles by Spearman’s rank correlation coefficient; only scores obtained by matching a number of measures (SpCs > 0) per protein pair greater than 50% of the subjects (per group), and with *p* ≤ 0.05, were considered. In addition, the same subset of proteins (*n* = 41, identification frequency = 100% per group) was processed to evaluate how the correlation changes between protein pairs in C, ALκ and ALλ groups.

### 2.4. Topological Analysis of PPI and Co-Expression Network Models

#### 2.4.1. Hubs Selection

Both reconstructed PPI and co-expression models were analyzed at topological level by Centiscape Cytoscape’s APP [39], as previously reported [40]. Diameter, average distance, degree, betweenness, centroid, stress, eigenvector, bridging, eccentricity, closeness, radiality and edge centralities were calculated for ppi network models, whereas diameter, average distance and degree were calculated for co-expression models. As for PPI models, nodes with both betweenness and centroid values above the average were considered hubs, whereas co-expression hubs were selected based on degree [22]. Statistical significance of topological results was tested by randomized network models [41]; *n* = 1000 random models per group were reconstructed and analyzed by *in-house* R scripts based on VertexSort (to build random models), igraph (to compute centralities) and ggplot2 (to plot results) libraries.

#### 2.4.2. Topological and Functional Modules Selection

CytoCluster Cytoscape’s APP [42] and ClusterONE algorithm were used to extract protein co-expression modules (*p* ≤ 0.01) from C, ALκ and ALλ models reconstructed from proteins identified in all subjects (*n* = 41, identification frequency = 100%); minimum size = 3, minimum density = “auto” and edge weights = Spearman’s rank correlation coefficient were set. Network models reconstructed by matching PPI (“databases”-annotated PPIs, score ≥ 0.3; “experiments”-annotated PPIs, score ≥ 0.15) and co-expression (Spearman’s rank correlation coefficient ≥ |0.5|, *p* ≤ 0.05) networks were analyzed by Community Detection Cytoscape’s APP [43]; HiDeF 1.1 beta algorithm (maximum resolution parameter = 25, consensus threshold = 75, persistent threshold = 5), while functional enrichment (reactome pathways) of the selected topological modules was performed by Enrichr algorithm (*p* ≤ 0.001, module size ≥ 4). Finally, the correlation change among subunits/proteins physically and functionally interacting in complexes and biological processes was evaluated in C, ALκ and ALλ groups.

### 2.5. Sub-Cutaneous Adipose Tissue Histological Analysis and TUBB4 Validation

Paraffin-embedded sections of sub-cutaneous adipose tissue were used for histological analysis. Briefly, after some preparation steps, the samples were incubated with rabbit anti-beta tubulin IV (TUBBIV; Ab179504, Abcam, Cambridge, UK) antibody. A Leica SP5 laser scanning confocal microscope (Leica Microsystem, Wetzlar, Germany) was used to acquire labeled samples. Finally, the images were analyzed using Image J software. Statistical analysis was carried out using Prism 8 (GraphPad Software, La Jolla, CA, USA). Data are presented as mean ± standard deviation (SD). Differences between sample means were evaluated with Student’s *t*-test (*p* ≤ 0.05). For major details, see Supplementary Material.

## 3. Results

### 3.1. Similarities and Differences in Amyloid-Deposition-Induced Proteome Remodeling in Abdominal Subcutaneous Adipose Tissue from ALκ and ALλ Patients

**Figure 1:**
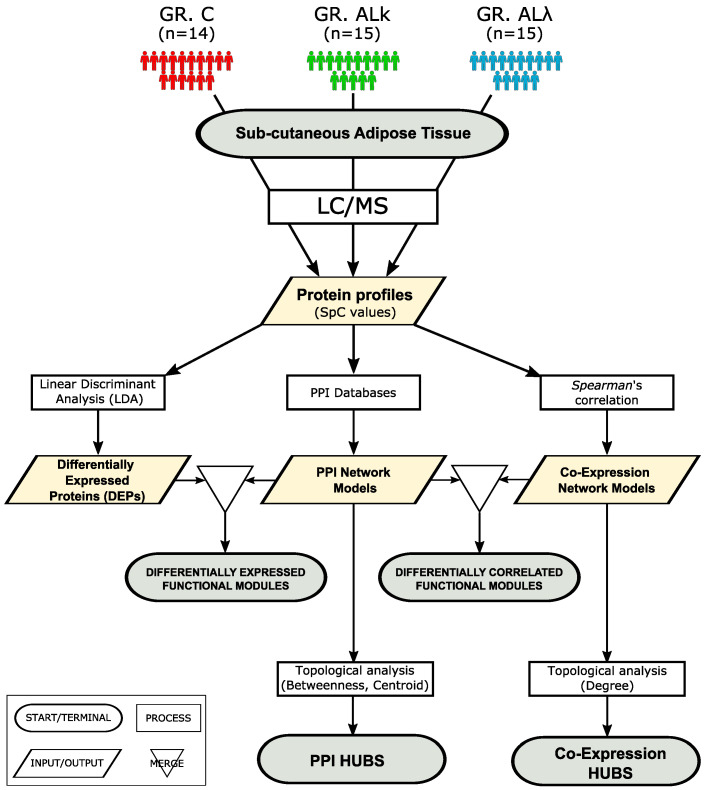
Workflow applied to investigate, at the proteomic system level, abdominal sub-cutaneous adipose tissues from control subjects (C, *n* = 14) and patients affected by ALκ (ALκ, *n* = 15) and ALλ (ALλ, *n* = 15) amyloidosis.

**Figure 2:**
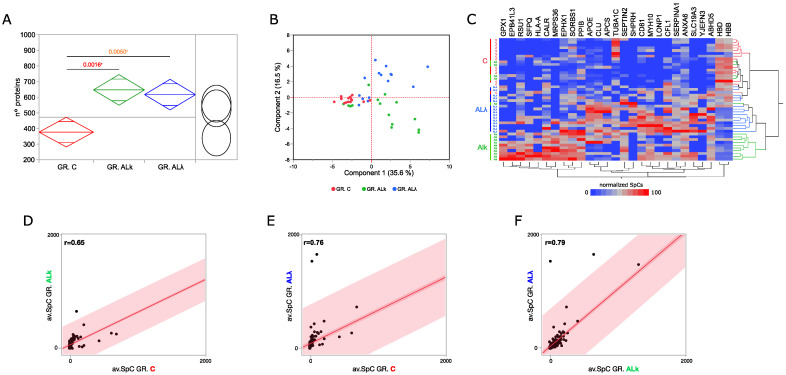
Proteomics analysis of abdominal sub-cutaneous adipose tissues from control subjects and patients affected by AL amyloidosis. (**A**) Comparison among the average number of proteins identified in control subjects (C, *n* = 14) and patients affected by AL amyloidosis (ALκ, *n* = 15; ALλ, *n* = 15); ANOVA and Tukey’s test (* *p* ≤ 0.05). (**B**) Principal component analysis (PCA) by processing proteins differentially abundant (DAPs) in C, ALκ and ALλ groups. (**C**) Hierarchical clustering and heat map showing the high-confidence DAPs in C, ALκ and ALλ protein profiles (LDA, *p* ≤ 0.01). (**D**) Spearman’s correlation using DAPs and the corresponding average SpC values in C and ALκ, (**E**) C and ALλ, and (**F**) ALκ and ALλ groups.

A set of protein profiles previously published [7,17,18,33], and collected by analyzing the abdominal subcutaneous adipose tissue of control subjects (C) and patients affected by ALκ and ALλ amyloidosis, was reprocessed following the workflow shown in (Figure 1). Compared to the control group, ALκ and ALλ patient profiles were characterized by a higher number of proteins (Figure 2A). This observation, which might have a link with an altered protein homeostasis [44], was associated with an enrichment in protein synthesis and metabolism we found in both AL patient’s groups, along with the mitochondrial respiration chain, lipid metabolism and immune system (Supplementary Figure S1). In ALκ and ALλ, we also found an enrichment in the amyloid fiber formation pathway [45]. Conversely, processes related to microtubules and ECM were less represented, and were most significant for ALκ tissues.

Following a deeper analysis of the enrichment results, 132 out of 4319 total identified proteins resulted in being differentially abundant (DAPs) (Supplementary Tables S1 and S2). In addition, to confirm previously published DAPs [18], new ones were extracted thanks to a larger cohorts of subjects. Although there were some exceptions, the set of higher-confidence DAPs discriminated the investigated groups of subjects (Figure 2B,C) well. As expected, the correlation between AL groups was higher (r = 0.79) than that observed with respect to C (Figure 2D–F). This value pointed out some slight differences in the modulation of the proteome when affected by the ALκ or ALλ isotype. These variations, and those identified in the comparison against control tissues, were classified and represented through 25 PPI functional modules (Supplementary Figure S2). Besides what has already been shown by the enrichment analysis (Supplementary Figure S1), in ALκ and ALλ, we observed the up-regulation of proteins involved in proteolysis, protein folding and REDOX homeostasis, while a small set of proteins involved in blood coagulation was down-regulated. Moreover, focusing on the comparison between AL groups, immune system and keratin-related proteins were more abundant in ALλ, whereas ALκ tissues showed a higher abundance of glutathione metabolism, protein synthesis and vesicle-transport-related proteins.

Among the most confident differentially abundant proteins (*p* ≤ 0.01), we found glutathione peroxidase 1 (GPX1), an important antioxidant enzymes that has already been described as protective in various neurodegenerative disorders, including Parkinson’s and Alzheimer’s disease [46]. It was identified in 80% and 47% of ALκ and ALλ tissues, respectively, whereas it was never found in the control group. Similarly, lon peptidase 1 (LONP1) and SNF2 histone linker PHD RING helicase (SHPRH) were mainly expressed in ALλ tissues (53% and 67%, respectively), and erythrocyte membrane protein band 4.1 Like 3 (EPB41L3) was mainly expressed in ALκ ones (53%). A further interesting protein was the CD81 antigen (CD81), which was more present in both patient groups (C IF = 36%, ALκ IF = 73%, ALλ IF = 93%). On the other hand, tubulin alpha-1C chain (TUBA1C) and hemoglobin subunit delta (HBD) were among the proteins that were less abundant in both ALκ and ALλ.

### 3.2. Protein–Protein Interaction (PPI) and Co-Expression Network Models Provide New Insights to Shed Light on Pathogenic Mechanisms Driven by Aggregation of ALκ and ALλ Isotypes

#### 3.2.1. PPI and Co-Expression Hubs Characterizing C, ALκ and ALλ Network Models

To exploit the correlation between a protein network’s structure and the functions that it supports, we transformed controls and patients protein profiles in PPI and protein co-expression network models. Following their topological analysis, the greatest differences concerned the average degree, which was higher in both AL cohorts. No notable centrality differences were observed at network level; the diameter and average distance were comparable in both PPI and co-expression models, even though the ALκ co-expression model had lower values that, combined with the high node degree, suggest a greater network compactness (Table 1 and Supplementary Table S3).

**Figure 3:**
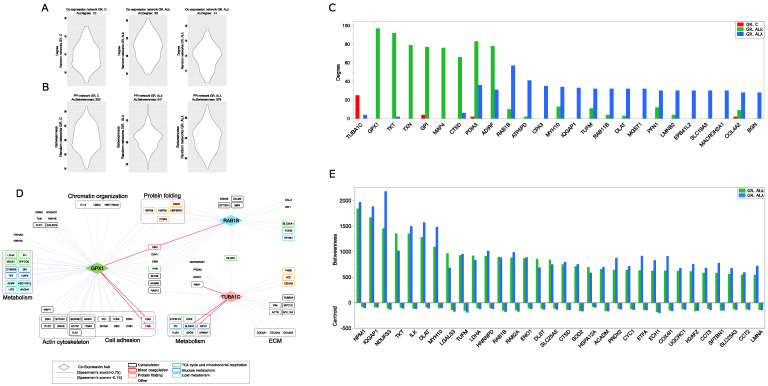
Protein-protein interaction (PPI) and co-expression network topology. (**A**) Validation of co-expression hubs. Violin plot of average degree values calculated from random co-expression networks (*n* = 1000 per group); for each group of subjects, the average degree in the reference co-expression network is shown, whereas in (**B**), the validation of PPI hubs is shown. Violin plot of average betweenness values calculated from random PPI networks (*n* = 1000 per group); for each group of subjects, the average betweenness in the reference PPI network is shown. (**C**) High-confidence differentially co-expressed proteins (co-expression hubs) selected by comparing the node degree from C, ALκ and ALλ co-expression network models (degree > 2 × network average degree). (**D**) Best-ranked co-expression hubs (TUBA1C in C, GPX1 ALκ and RAB1B in ALλ) and their higher-confidence correlation partners (Spearman’s correlation score ≥$\left|0.85\right|$ for GPX1 and RAB1B, ≥$\left|0.75\right|$ for TUBA1C). (**E**) PPI hubs, selected by betweenness and centroid values, from both ALκ and ALλ PPI network models.

In order to go deeper into the selection of topologically relevant nodes, we extracted a set of PPI and co-expression network hubs, as putative key proteins involved in pathophysiological processes running in ALκ and ALλ tissues (Figure 3A,B). Regarding co-expression models, TUBA1C, GPX1 and ras-related protein Rab-1B (RAB1B) were the best ranked hubs in C, ALκ, ALλ models, respectively (Figure 3C,D and Supplementary Table S4). GPX1 resulted in being highly correlated with proteins involved in cell adhesion, while both GPX1 and RAB1B correlated with proteins involved in mitochondrial metabolism and protein folding. They showed a negative correlation with hemoglobin subunit delta (HBD), and GPX1 also had a similar connection with the fibrinogen gamma chain (FGG) and fibrinogen beta chain (FGB) (Figure 3D). Concerning the C group, a correlation between TUBA1C and collagen IV subunits emerged.

Other isotype-specific co-expression hubs included thioredoxin (TXN), microtubule associated protein 4 (MAP4) and Cathepsin D (CTSD) for ALκ, and ATP synthase subunit d (ATP5PD) and Mast cell carboxypeptidase A (CPA3) for ALλ (Figure 3C). TXN and CTSD were also ALκ PPI hubs, whereas RAB1B, myosin-10 (MYH10) and dihydrolipoamide S-acetyltransferase (DLAT) were simultaneously ALλ PPI and co-expression hubs (Figure 3C–E and Supplementary Tables S4 and S5). In this scenario, PPI models have consistently highlighted the role of mitochondria by a number of PPI hubs involved in mitochondrial metabolism and respiration (CYC1, DLAT, UQCRC1, NDUFS3, COX4I1, ACADM, ECH1, ETFA), oxidative stress (SOD2) and translation (TUFM). Along with them, another consistent set of PPI hubs featuring both ALκ and ALλ was involved in the unfolded protein response (NPM1, HSPA12A, CCT2, CCT3); (Figure 3E).

#### 3.2.2. PPI and Co-Expression Modules Affected by Aggregation of ALκ and ALλ Isotypes

**Figure 4:**
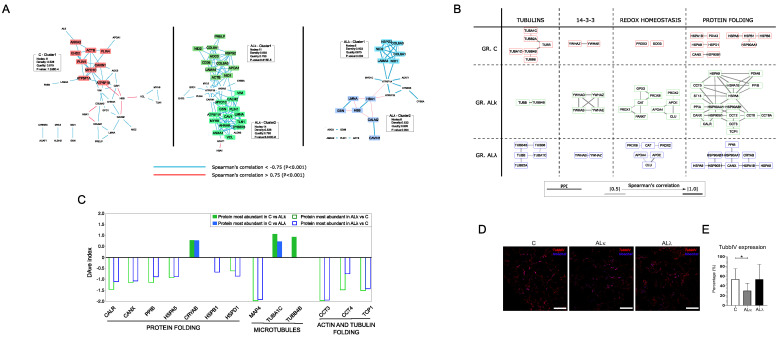
PPI functional modules differentially correlated. (**A**) Clusters of proteins highly correlated in C, ALκ and ALλ. (**B**) Correlation changes among subunits/proteins physically and functionally interacting in complexes and biological processes. (**C**) DAve index of DAPs involved in protein folding and microtubule-related processes. A positive DAve index indicates that protein most abundant in C, whereas negative DAve index indicates that protein most abundant in ALκ and ALλ. (**D**) Representative immunofluorescence images of sub-cutaneous adipose tissue from control subjects (**C**) and patients affected by AL amyloidosis (ALκ and ALλ) stained against beta Tubulin IV (TUBBIV, red). Nuclei were detected with Hoechst (blue). Scale bars represent 150 μm. (**E**) The graph highlights the quantification of TUBBIV in the immunostained samples; * *p* ≤ 0.05.

The combination of PPI and co-expression models was initially processed for the identification of a highly correlated interacting community of nodes. Most of them were enriched in pathways related to metabolism, cytoskeleton, transport and protein folding (Supplementary Figure S3). The latter two were mainly represented in ALκ and ALλ, along with communities enriched in the detoxification of reactive oxygen species. Communities enriched in extracellular matrix (ECM) organization processes were found in all groups. In this context, an interesting difference emerged in the co-expression network modules. In fact, in both ALκ and ALλ, a weighted cluster analysis evidenced the presence of the heparan sulfate proteoglycan core protein (HSPG2) in modules enriched by ECM proteins, whereas this did not happen in the C model (Figure 4A). This result could fit with the observation that heparan sulfate proteoglycans (HSPGs) are commonly found in amyloid deposits; therefore, they have been suggested to be functionally involved in the pathogenesis of amyloidosis [47].

Besides the analysis of node communities, the merging of the PPI and co-expression models was performed with the aim of clarifying the question of whether proteins that are part of protein complexes and/or biological processes are also correlated in maintaining a well-defined stoichiometry. Therefore, the question of how to interpret the correlation loss or gain arises. Following these tracks, we spotted some protein complexes whose correlation changed from C to ALκ and ALλ groups (Figure 4B). In particular, the ALκ group showed a significant decrease in correlation among tubulins, whereas, in the ALλ group, the same phenomenon was observed for the 14-3-3 proteins complex. These variations were associated with an increased correlation of proteins physically interacting and functionally involved in the redox homeostasis/detoxification of reactive oxygen species and protein folding/stress response. Their increase was much more marked for the ALκ group, where the protein folding/stress response module was featured by the presence of different subunits belonging to the T-complex protein ring complex (TRiC). As TRiC is involved in tubulin folding, our attention was captured by the opposite trend of expression between its components (CCT3, CCT4, TCP1) and tubulin subunits (TUBA1C, TUBB4B) (Figure 4C–E and Supplementary Figure S2). In fact, this suggests a potential non-random correlation of events worthy of further future investigation. In addition, except for the alpha-crystallin B chain (CRYAB), other folding-related proteins were more abundant in ALκ and ALλ tissues, confirming a role of the endoplasmic reticulum (ER) in systemic amyloid diseases [48].

## 4. Discussion

To our knowledge, our perspective study represents the first example of an investigation of systemic amyloidosis through methods based on graph theory applied to proteomic data from the analysis of complex human tissues. Due to the small number of subjects, which does not allow for more robust stratifications, the results that we extracted represent the mean emerging from groups created following the presence (or absence in healthy controls) of amyloidogenic AL chains in subcutaneous adipose tissue. However, as previously reported by Brambilla et al. [33], most patients were further united by heart and kidney involvement.

Globally, the observations collected here draw a landscape where ALκ and ALλ tissues undergo a similar proteome modulation. In addition, slight and interesting differences have been noted. These findings are consistent with those previously reported by our group [18]. Thanks to a larger cohorts of subjects, new differentially abundant proteins were extracted, while ECM/cytoskeleton, proteostasis and mitochondrial-related processes being confirmed as those most affected by amyloid deposition in subcutaneous adipose tissues.

Going deeper through network approaches, here, we selected new proteins that could be central in the pathophysiological processes underlying AL amyloidosis. A case in point concerns proteins and pathways involved in oxidative stress. As reported for cultured cells from multiple target tissues, there is solid evidence that AL-induced toxicity is associated with an increase in ROS and mitochondrial alterations [49,50,51,52,53,54]. In our tissues, the relevance of GPX1 in terms of protein expression and the network hub fits with its role in contrasting ROS-mediated toxic effects, as already demonstrated in Parkinson’s and Alzheimer’s disease [46]. A scenario of oxidative stress is further suggested by other hubs, such as SOD2, TXN and Peroxiredoxin-2 (PRDX2). It is worth noting that we found a marked negative correlation between GPX1 and some proteins, such as FGB, FGG and HBD, related to hemostasis and blood coagulation. This observation drew our attention to a potential relationship between oxidative stress and abnormal bleeding and fibrinolysis observed in AL patients [55]. Although this correlation has previously been reported in patients with isolated aortic stenosis [56], no evidence is currently available in patients with amyloidosis, but it could represent an interesting hypothesis to be explored in future studies.

Oxidative stress, mitochondrial dysfunction and apoptosis represent interrelated processes known to occur in experimental models of AL amyloidosis [51,54], as well as neurodegenerative diseases [57]. The centrality of the mitochondrion has been clearly shown in our study by a number of DAPs and PPI hubs characterizing ALκ and ALλ models. Among the high-confidence DAPs, we noticed LONP1, a mitochondrial protease that mediates the selective degradation of misfolded, unassembled or oxidatively damaged polypeptides [58]; it also works as a molecular chaperone and cooperates with heat shock 70 kDa protein 1B (HSPA1B) to promote mitochondrial protein folding and contrasting cell death in response to oxidative stress [59]. Proteoastasis-related proteolytic processes were also correlated with the abundance and topological relevance of other proteases found in our profiles, including CTSD. As a matter of fact, it has been described as physiologically important in serum amyloid A (SAA) degradation in amyloidosis [60], while different studies have established its role in the process of autophagy in neurodegenerative diseases [61].

The number of DAPs, hubs and modules enriched in heat shock proteins/chaperones could support the activation of the protein quality control (PQC) systems [62], as well as the implication of the endoplasmic reticulum (ER) in systemic amyloid diseases [48]. In this context, the recurrence of some proteins belonging to the T-complex protein ring complex (TRiC) is noteworthy [63]. TRiC is an essential and ubiquitous component of the protein-folding machinery of eukaryotic cells. It has been described as both a potential modulator of protein aggregation [64] and neuroprotective factor in Huntington’s disease by inhibiting the aggregation of the mutant huntingtin [65]. Moreover, it is also required during sarcomere assembly in myofibers [66] and for the folding of abundant cytoskeletal proteins, such as actin and tubulin [64]. This last function might have a link with the decrease in correlation among tubulin subunits that we mainly observed in the ALκ group. The greater expression and topological relevance of the TRiC complex subunits could support a potential tubulin stress, which is an effect already described as a common feature of many neurodegenerative diseases [67]. A putative influence of amyloid deposition on microtubules was further suggested by the topological relevance of MAP4 in ALκ and its up-regulation in both ALκ and ALλ groups. MAP4 is a major non-neuronal microtubule-associated protein belonging to the MAP2 and TAU family. In addition to regulating organelle transport along the cytoskeletal microtubules, it is involved in maintaining mitochondrial homeostasis, and it has been proposed as a potential candidate in multiple cardiovascular pathologies [68].

Finally, unlike in ALκ, a significant and interesting decrease in correlation in the ALλ model was observed for 14-3-3 proteins, a complex that plays a pivotal role in cellular signal transduction, counting more than 200 interactors [69]. Several studies have shown that 14-3-3 acts as a molecular adaptor to recruit chaperone-associated misfolded proteins to dynein motors for transport to aggresomes [70]. More recently, the activity of specific 14-3-3 subunits as molecular chaperones has also been demonstrated, such as 14-3-3σ transiently interacting with amyloid β (Aβ) in vitro and inhibiting fibril formation [71], and 14-3-3η interacting with human α-synuclein aggregation intermediates, reducing their cellular toxicity [72].

## 5. Conclusions

Looking at the proteome modulation from different points of view, including the network topology, appeared to be a promising approach for ranking protein candidates that may play a key role in pathophysiological mechanisms induced and affected by amyloid deposition, respectively. Many targets related to the cytoskeleton, oxidative stress and mitochondrial dysfunction overlap with molecules previously described in other protein-misfolding diseases, such as Alzheimer’s, Parkinson’s and Huntington’s diseases. Although we are well aware of the differences that distinguish amyloidosis from more common neurodegenerative diseases, this matching could be construed as a virtual validation of our findings. At the same time, they could be indicative of the aggregation of unfolded proteins inducing similar mechanisms regardless of the amyloid protein and tissues/organs targeted, and thus an overlapping that would allow us to speculate on the use of adipose tissue as a mirror to infer potential molecular events occurring in other sites, including the heart.

The number of subjects, as well as their retrospective analysis, represents one of the major limitations of our study. Although all samples were processed under the same protein extraction protocol, it is undeniable that they were analyzed using different MS instruments and methods; however, we processed our data to minimize potential batch effects and to extract the most robust and meaningful information. Also in light of this, rather than drawing conclusions, our findings represent a source of information to design new target experiments. On the other hand, our study is a proof-of-concept for future applications that will take into account a larger cohort of patients and different organs/tissues affected. This goal brings attention to the need for collaborative and multicenter studies. With the support of single-cell technologies, they would allow for a better clinical stratification, which, in turn, would improve the association with molecular data, favoring the identification of targets for diagnostic, prognostic and therapeutic purposes, as well as helping to shed light on as yet unanswered questions, such as disparities in organ damage severity, organ response to treatments and even organ tropism.

## Figures and Tables

**Table 1:** Network centralities from PPI and co-expression models.

**PPI Model**	**Nodes**	**Edges**	**Diameter**	**Av. Distance**	**Degree**
C	256	2757	5	2.38	21.5
ALκ	400	5644	5	2.35	28.2
ALλ	407	5315	5	2.42	26.1
**Co-Expression Model**	**Nodes**	**Edges**	**Diameter**	**Av. Distance**	**Degree**
C	227	1212	7	2.97	10
ALκ	361	5438	6	2.52	30
ALλ	350	2534	9	2.88	14

## Data Availability

The proteomic datasets (in form of raw data) analyzed for this study are available (3 January 2023) in the MassIVE database (massive.ucsd.edu) under the ID: MSV000090875, or by ftp at link ftp://massive.ucsd.edu/MSV000090875/.

## Supplementary Materials

The following supporting information can be downloaded at: https://www.mdpi.com/article/10.3390/cells12050699/s1, Figure S1: Reactome pathways differentially enriched in Control, ALκ and ALλ protein profiles; Figure S2: Protein-protein interaction (PPI) functional modules differentially expressed in Control, ALκ and ALλ; Figure S3: Protein-protein interaction (PPI) and Co-Expression node communities enriched in Control, ALκ and ALλ network models; Figure S4: Protein-protein interaction (PPI) hubs specifically found in Control, ALκ and ALλ; Table S1: Protein profiles from abdominal subcutaneous adipose tissue of control subjects and patients affected by AL amyloidosis; Table S2: Differentially abundant proteins (DAPs) by comparing Control, ALκ and ALλ profiles; Table S3: Centralities average values from Control, ALκ and ALλ protein-protein interaction (PPI) network models; Table S4: Co-expression network hubs; Table S5: Protein-protein interaction (PPI) network hubs.

## Abbreviations

The following abbreviations are used in this manuscript:

AL	Immunoglobulin light chain
ALκ	Patients affected by amyloidosis κ
ALλ	Patients affected by amyloidosis λ
C	Control subjects
DAPs	Differentially abundant proteins
DAve	Differential average
DEGs	Differentially expressed genes
FDR	False discovery rate
HSPGs	Heparan sulfate proteoglycans
MS	Mass spectrometry
LDA	Linear discriminant analysis
PCA	Principal component analysis
PPI	Protein–protein interaction
SD	Standard deviation
SpC	Spectral count
