# Supplementary material for: The Protein Network in Subcutaneous Fat Biopsies from Patients with AL Amyloidosis: More Than Diagnosis?
Source: Cells. 2023 Feb 22;12(5):699. doi: 10.3390/cells12050699 (PMC10000381; doi:10.3390/cells12050699)
Supplement: Supplementary file 1 [file cells-12-00699-s001.zip › Supplementary files/template SI.pdf]

Supplementary Materials: The protein network in subcutaneous fat biopsies from patients with AL amyloidosis, more than diagnosis?

Dario Di Silvestre [1,*], Francesca Brambilla [1], Francesca Lavatelli [2,11], Maila Chirivì [3,10], Diana Canetti [5], Claudia Bearzi [1,6], Roberto Rizzi [4,6], Johan Bijzet [7,8], Bouke Pier Hazenberg [7,8], Vittorio Bellotti [5,9], Julian D. Gillmore [5] and Pierluigi Mauri[1,*]

1. Materials and Methods

1.1. Proteomic analysis and raw data processing

Abdominal subcutaneous adipose tissue protein profiles previously collected were re-analyzed and used as reference dataset [1–4]. The considered samples were collected and analyzed over a period of time ranging from 2008 to 2015, and by means of three different MS instruments, such as LTQ (coupled with bidimensional liquid chromatography), LTQ ORBITRAP XL (coupled with bidimensional liquid chromatography) and QExactive HF (coupled with monodimensional liquid chromatography) mass spectrometers (see Table). All analyses were performed at Institute for Biomedical Technologies-National Research Council (ITB-CNR), Segrate (Milan), Italy, where they were processed using the same extraction protocol. As reported by Brambilla et al. [1], most of the considered samples showed Congo Red score $\geq 3+$, and they were mainly characterized by heart and kidney involvement (data not shown).

Samples, MS instruments and n° technical replicates								
Group C	Instrument	n° rep	Group ALκ	Instrument	n° rep	Group ALλ	Instrument	n° rep
C1	LTQ	1	ALκ1	LTQ	2	ALλ1	LTQ	2
C2	LTQ	2	ALκ2	QEXA	2	ALλ2	LTQ	2
C3	LTQ	2	ALκ3	QEXA	2	ALλ3	LTQ	2
C4	LTQ	2	ALκ4	QEXA	2	ALλ4	LTQ	2
C5	LTQ	2	ALκ5	QEXA	2	ALλ5	LTQ	2
C6	LTQ	2	ALκ6	QEXA	2	ALλ6	LTQ	2
C7	LTQ	2	ALκ7	QEXA	2	ALλ7	LTQ	2
C8	LTQ	1	ALκ8	ORBITRAP	1	ALλ8	QEXA	2
C9	QEXA	1	ALκ9	ORBITRAP	1	ALλ9	QEXA	2
C10	LTQ	2	ALκ10	ORBITRAP	1	ALλ10	QEXA	2
C11	LTQ	2	ALκ11	ORBITRAP	1	ALλ11	QEXA	2
C12	QEXA	2	ALκ12	ORBITRAP	1	ALλ12	ORBITRAP	1
C13	LTQ	1	ALκ13	ORBITRAP	1	ALλ13	LTQ	1
C14	LTQ	2	ALκ14	ORBITRAP	1	ALλ14	LTQ	1
			ALκ15	QEXA	1	ALλ15	LTQ	1

1.1.1. LTQ

Trypsin-digested adipose tissue samples were analyzed by Multidimensional Protein Identification Technology (MudPIT), using a Proteome 2X system (Thermo Fisher Scientific, San Jose, CA, USA). Peptide mixtures were separated by strong cation exchange chromatography onto an SCX column (Biobasic-SCX column, 0.32 i.d. × 100 mm, 5 m, ThermoHypersil, Bellofonte, PA, USA). Fractions were eluted stepwise with a nine-step ammonium chloride concentration gradient (0, 20, 40, 80, 120, 200, 400, 600, 700 mM NH4Cl). Each salt step eluate was captured onto C18 traps for concentration and desalting, prior to final separation on the capillary reversed phase column (Biobasic-C18, 0.180 i.d. ×100 mm, 5 μm particle size, Thermo Fisher Scientific, San Jose, CA, USA). Peptides were eluted using an acetonitrile gradient ((A) 0.1% formic acid in water; (B) 0.1% formic acid in acetonitrile); the gradient profile was 5% eluent B for 5 min, 565% B in 50 min, 65% B for 3 min, and 6595% B in 10 min. The flow rate on C-18 column was 1 μL/min. The peptides eluted from the C18 column were directly analyzed with an LTQ mass spectrometer (Thermo Fisher Scientific, San Jose, CA, USA) equipped with a nano-ESI source. Full MS spectra were acquired in positive mode over a 4002000 m/z range, followed by five MS/MS events sequentially generated in a data-dependent manner on the first five most intense ions selected from the full MS spectrum (collision energy 35%) and using dynamic exclusion for MS/MS analysis.

1.1.2. LTQ ORBITRAP

Trypsin-digested adipose tissue samples were analyzed by Multidimensional Protein Identification Technology (MudPIT), using a Proteome 2X system (Thermo Fisher Scientific, San Jose, CA, USA). Peptide mixtures were separated by strong cation exchange chromatography onto an SCX column (Biobasic-SCX column, 0.32 i.d. × 100 mm, 5 μm, ThermoHypersil, Bellofonte, PA, USA). Fractions were eluted stepwise with a nine-step ammonium chloride concentration gradient (0, 20, 40, 80, 120, 200, 400, 600, 700 mM NH4Cl). Each salt step eluate was captured onto C18 traps for concentration and desalting, prior to final separation on the capillary reversed phase column (Biobasic-C18, 0.180 i.d. ×100 mm, 5 μm particle size, Thermo Fisher Scientific, San Jose, CA, USA). Peptides were eluted using an acetonitrile gradient ((A) 0.1% formic acid in water; (B) 0.1% formic acid in acetonitrile): the gradient profile was 5% eluent B for 3 min, 5–40% B in 50min, 40–80% B in 10min, 80–95% B in 5min, 95% B in 10min. The flow rate on C-18 column was 2 μl/min. Then, eluting peptides were electro-sprayed directly into a hybrid ion trap-Orbitrap mass spectrometer (LTQ Orbitrap XLTM ETD; Thermo Fisher Scienti!c, Inc.), equipped with a nano-spray ion source. The spray capillary voltage was set at 1.5kV, and the ion transfer capillary temperature was maintained at 220 °C. For each step of peptide elution from C18 column, full mass spectra were recorded in the positive ion mode over a 400–1600 m/z range, with a resolving power of 60,000 (full width at half-maximum) and a scan rate of 2 spectra/s. This step was followed by four low-resolution MS/MS events that were sequentially generated in a data-dependent manner on the top four ions selected from the full MS spectrum, using dynamic exclusion for the MS/MS analysis.

1.1.3. QExactive

Trypsin-digested mixtures were analyzed by the Eksigent nanoLC-Ultra 2D System (Eksigent, AB SCIEX Dublin, CA, USA) combined with cHiPLC- nanoflex system (Eksigent) in trap-elute mode on a nano cHiPLC column (75 mm 15 cm ChromXP C18-CL, 3 mm, 120 Å), through a 65 minute gradient of 5–45% of eluent B (eluent A, 0.1% formic acid in water; eluent B, 0.1% formic acid in acetonitrile), at a flow rate of 300 nL/min. MS spectra were acquired using a QExactive mass spectrometer (Thermo Fisher Scientific, San Jose, CA, USA), equipped with an EASY-Spray ion source (Thermo Fisher Scientific). Easy spray was achieved using an EASY-Spray Emitter (Dionex Benelux BV, Amsterdam, The Netherlands) (nanoflow 7 μm ID Transfer Line 20 μm × 50 cm) held to 2.1 kV, while the ion transfer capillary was held at 220 °C. Full mass spectra were recorded in positive ion mode over a 400 to 1600 m/z range with a resolution setting of 70,000 FWHM (Full Width at Half Maximum) (m/z 200) with 1 microscan per second. Each full scan was followed by 7 MS/MS events, acquired at a resolution of 17,500 FWHM, sequentially generated in a data dependent manner on the top seven most abundant isotope patterns with charge $\geq$2, selected with an isolation window of 2 m/z from the survey scan, fragmented by higher energy collisional dissociation (HCD) with normalized collision energies of 30 and dynamically excluded for 30 sec. The maximum ion injection times for the survey scan and the MS/MS scans were 50 and 200 ms and the ion target values were set to 10^6 and 10^5, respectively.

1.2. MS/MS data processing

The corresponding raw data were here reprocessed by the SEQUEST HT algorithm and Proteome Discoverer 2.5 software (Thermo Fisher Scientific, CA, USA). Experimental MS/MS spectra were compared with the theoretical mass spectra obtained by *in silico* digestion of *Homo sapiens* protein sequences downloaded from UNIPROT (www.uniprot.org) in December 2021. The following criteria of searching were set: trypsin enzyme, three missed cleavages per peptide, mass tolerances on precursor ions was set to ± 500, 100 and 50 ppm for LTQ, Orbitrap and QExactive analyses, respectively, while ± 0.8 Da were set for fragment ions. Percolator node was used with a target-decoy strategy to give a final false discovery rates (FDR) $\leq$0.01 (strict) based on q-values, considering maximum deltaCN of 0.05. Only peptides with minimum peptide length of six amino acids, confidence at "High" level and rank 1 were considered. Protein grouping and strict parsimony principle were applied.

1.3. Label-free quantitative analysis: pairwise comparisons and DAve index

Pairwise comparisons (C *vs* ALκ; C *vs* ALλ and ALκ *vs* ALλ) were evaluated by DAve index:

$$(SpC_{\mathrm{A}} - SpC_{\mathrm{B}})/(SpC_{\mathrm{A}} + SpC_{\mathrm{B}})/0,5$$

where A and B represent the conditions compared); conventionally, the DAve index of proteins exclusively identified only in one of the conditions under comparison was set to $\pm$2. Specifically, positive DAve values indicate proteins up-regulated in A (and down-regulated in B), while negative DAve values indicate proteins up-regulated in B (and down-regulated in A); -0.4$\geq$DAve$\geq$0.4.

1.4. Sub-cutaneous adipose tissue histological analysis and TUBB4 validation

Briefly, paraffin-embedded sections were dewaxed, rehydrated in a descending alcohol series and subjected to antigen retrieval procedure using citrate buffer pH 6.0. The slides were washed with 1× PBS and blocked with 5% bovine serum albumin (BSA, Sigma-Aldrich) for 30 min at room temperature (RT) to saturate non-specific binding sites. Then, the samples were incubated with rabbit anti-beta tubulin IV (TUBBIV; Ab179504, Abcam) antibody diluted 1:150 in 0.5% BSA solution overnight at 4 °C. After washing with 1× PBS, the slides were incubated for 1 hour at 37 °C with goat anti-rabbit Alexa Fluor568 (Invitrogen; dilution 1:1000) secondary antibody. Nuclei were counterstained with Hoechst for 30 min and washed twice with 1× PBS. A Leica SP5 laser scanning confocal microscope (Leica Microsystem, Wetzlar, Germany) was used to acquire labeled samples. Globally, we evaluate only 2 subjects per group, and 3 sections per subject. The images were analyzed using Image J software. The images were converted into black and white in the order listed below: Process>Binary>Convert to Binary. Then, tubulin expression was evaluated using the Analyze>Analyze Particles command by obtaining the area measurement of positive particles.

1.5. Figures, Tables and Schemes

Abbreviations

The following abbreviations are used in this manuscript:

ALκ	Patients affected by amyloidosis κ
ALλ	Patients affected by amyloidosis λ
BSA	Bovine Serum Albumin
C	Control subjects
DAve	Differential Average
ESI	Electrospray Ionization
ETD	Electron-transfer dissociation
FDR	False Discovery Rate
FWHM	Full Width at Half Maximum
HCD	Higher Collisional Dissociation
LDA	Linear Discriminant Analysis
MS	Mass spectrometry
MS/MS	Tandem Mass Spectra
MudPIT	Multidimensional Protein Identification Technology
PBS	Phosphate-Buffered Saline
PPI	Protein-Protein Interaction
RT	Room Temperature
SCX	Strong Cation Exchange
SpC	Spectral count

References

1. Brambilla, F.; Lavatelli, F.; Di Silvestre, D.; Valentini, V.; Rossi, R.; Palladini, G.; Obici, L.; Verga, L.; Mauri, P.; Merlini, G. Reliable typing of systemic amyloidoses through proteomic analysis of subcutaneous adipose tissue. *Blood* **2012**, *119*, 1844–1847. https://doi.org/10.1182/blood-2011-07-365510.
2. Brambilla, F.; Lavatelli, F.; Valentini, V.; Di Silvestre, D.; Obici, L.; Mauri, P.; Merlini, G. Changes in tissue proteome associated with ATTR amyloidosis: insights into pathogenesis. *Amyloid : the international journal of experimental and clinical investigation : the official journal of the International Society of Amyloidosis* **2012**, *19 Suppl 1*, 11–13. https://doi.org/10.3109/13506129.2012.674989.
3. Brambilla, F.; Lavatelli, F.; Di Silvestre, D.; Valentini, V.; Palladini, G.; Merlini, G.; Mauri, P. Shotgun protein profile of human adipose tissue and its changes in relation to systemic amyloidoses. *Journal of proteome research* **2013**, *12*, 5642–5655. https://doi.org/10.1021/pr400583h.
4. Canetti, D.; Brambilla, F.; Rendell, N.B.; Nocerino, P.; Gilbertson, J.A.; Di Silvestre, D.; Bergamaschi, A.; Lavatelli, F.; Merlini, G.; Gillmore, J.D.; et al. Clinical Amyloid Typing by Proteomics: Performance Evaluation and Data Sharing Between Two Centres. *Molecules (Basel, Switzerland)* **2021**, *26*. https://doi.org/10.3390/molecules26071913.

Disclaimer/Publisher's Note: The statements, opinions and data contained in all publications are solely those of the individual author(s) and contributor(s) and not of MDPI and/or the editor(s). MDPI and/or the editor(s) disclaim responsibility for any injury to people or property resulting from any ideas, methods, instructions or products referred to in the content.

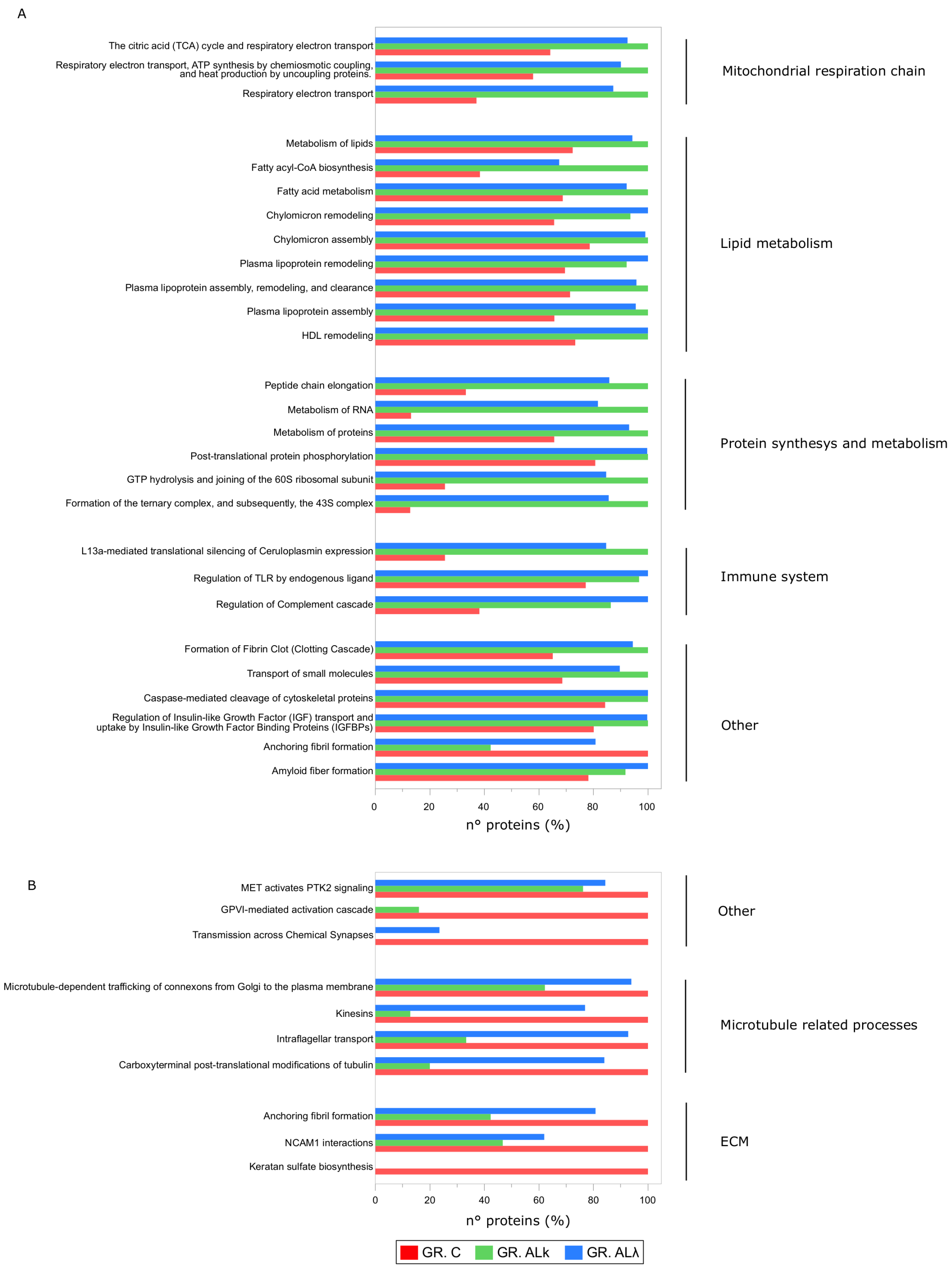

Figure S1. Reactome pathways differentially enriched by comparing the protein profiles obtained from abdominal sub-cutaneous adipose tissues of control subjects (C, n=14) and patients affected by AL amyloidosis (ALκ, n=15; ALλ n=15). **A)** Pathways most enriched in both ALκ and ALλ patients. **B)** Pathways most enriched in control subjects (C). Enriched Reactome pathways were retrieved by STRING Cytoscape's APP, and compared by Linear Discriminant Analysis (LDA) (P$\leq$ 0.01)

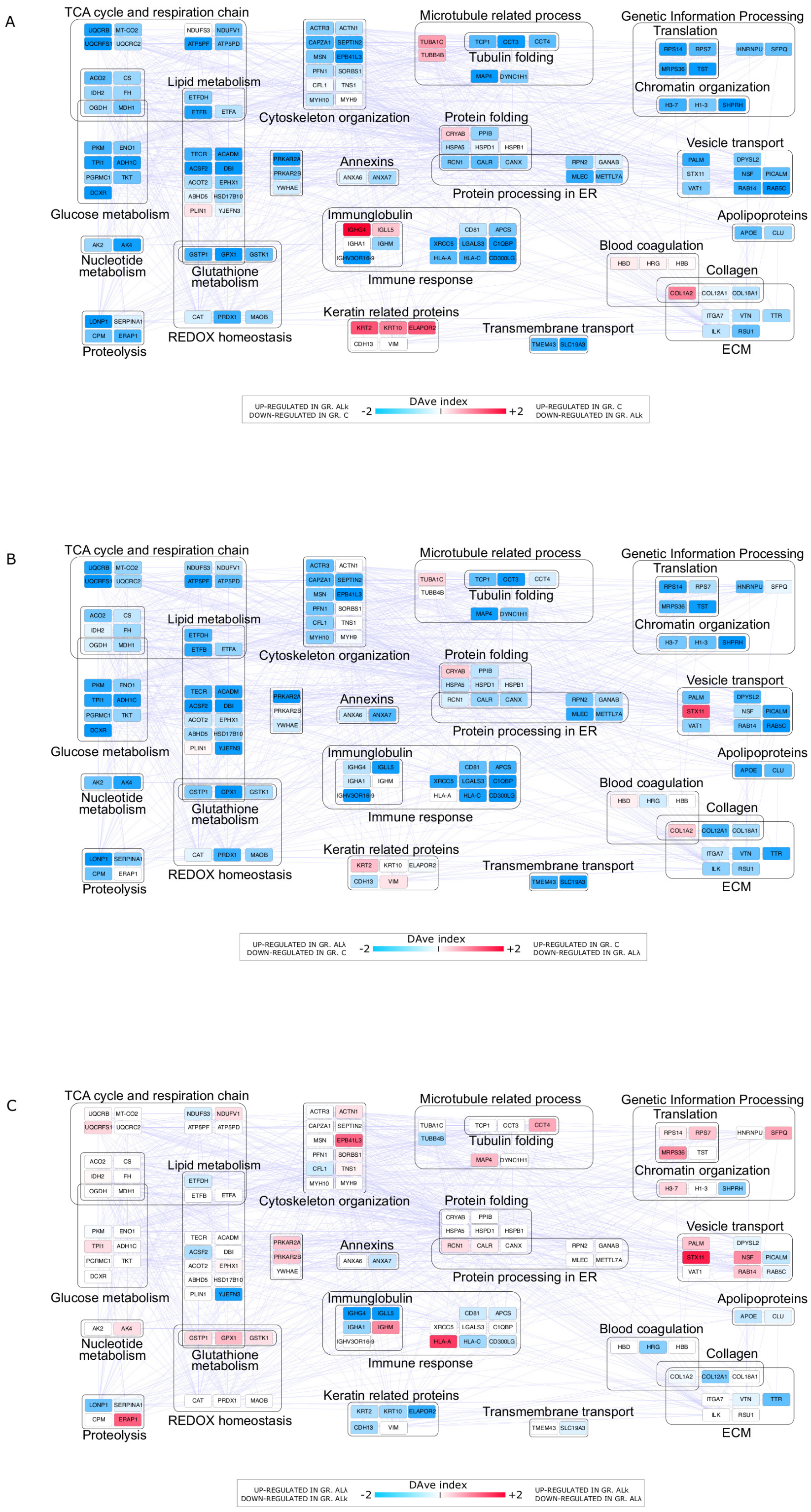

Figure S2. Protein-protein interaction (PPI) network and functional modules differentially enriched. The reconstructed network was visualized by Cytoscape, and in pairwise comparisons (**(A)** Control *vs* ALκ, **(B)** Control *vs* ALλ, **(C)** ALκ *vs* ALλ) the color code indicates up-regulated (red) and down-regulated (light blue) proteins based on the DAve index.

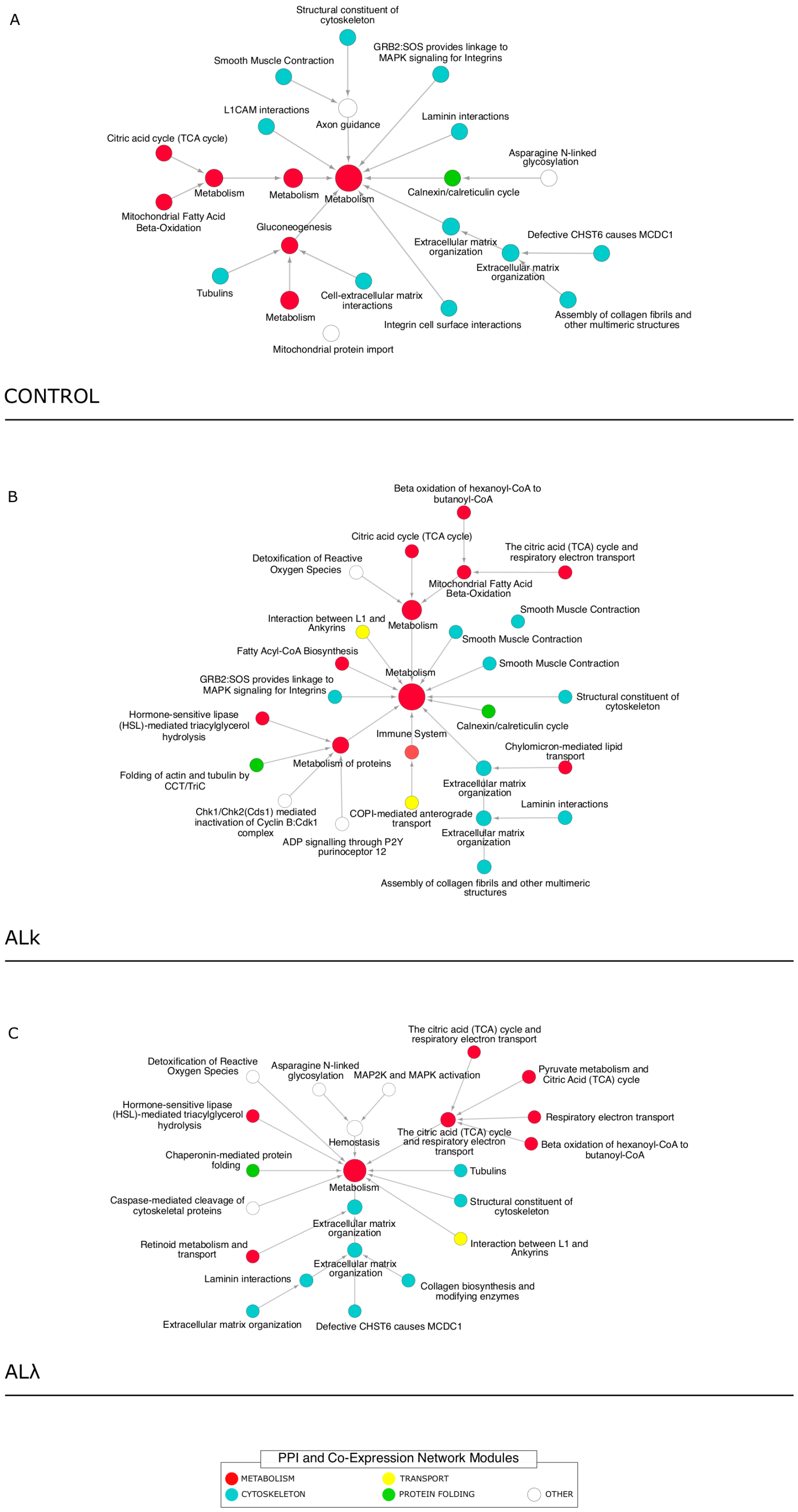

Figure S3. PPI and Co-Expression node communities enriched in **A)** Control, **B)** ALκ, **C)** ALλ network models; Community Detection Cytoscape's APP, HiDeF 1.1 beta algorithm and Enrichr algorithm were used (P$\leq$0.001, module size$\geq$4).

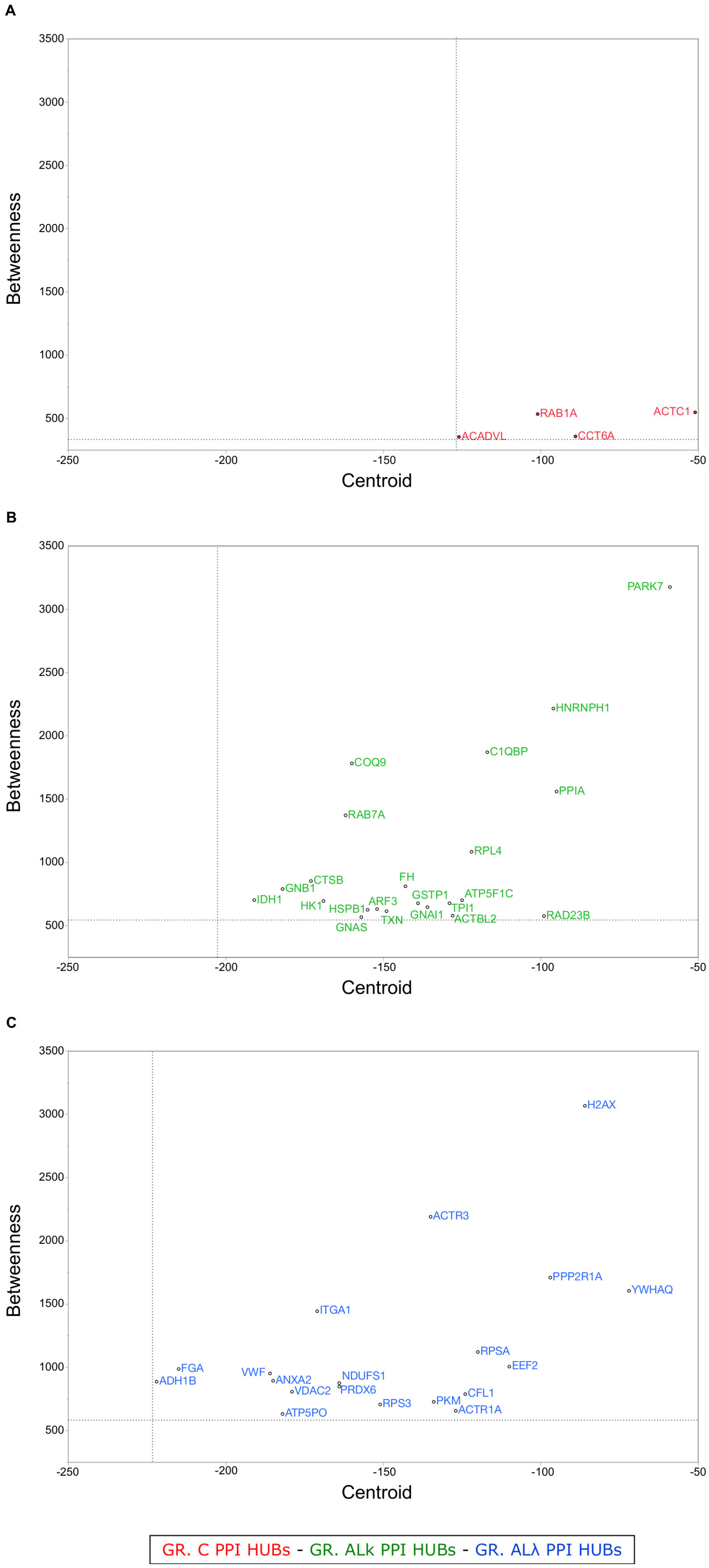

Figure S4. PPI hubs specifically found in **A)** C, **B)** ALκ and **C)** ALλ protein-protein interaction (PPI) network models. Dot lines indicate Betweenness and Centroid average values.
