# Supplementary figures and images for: The Protein Network in Subcutaneous Fat Biopsies from Patients with AL Amyloidosis: More Than Diagnosis?
Source: Cells. 2023 Feb 22;12(5):699. doi: 10.3390/cells12050699 (PMC10000381; doi:10.3390/cells12050699)

A

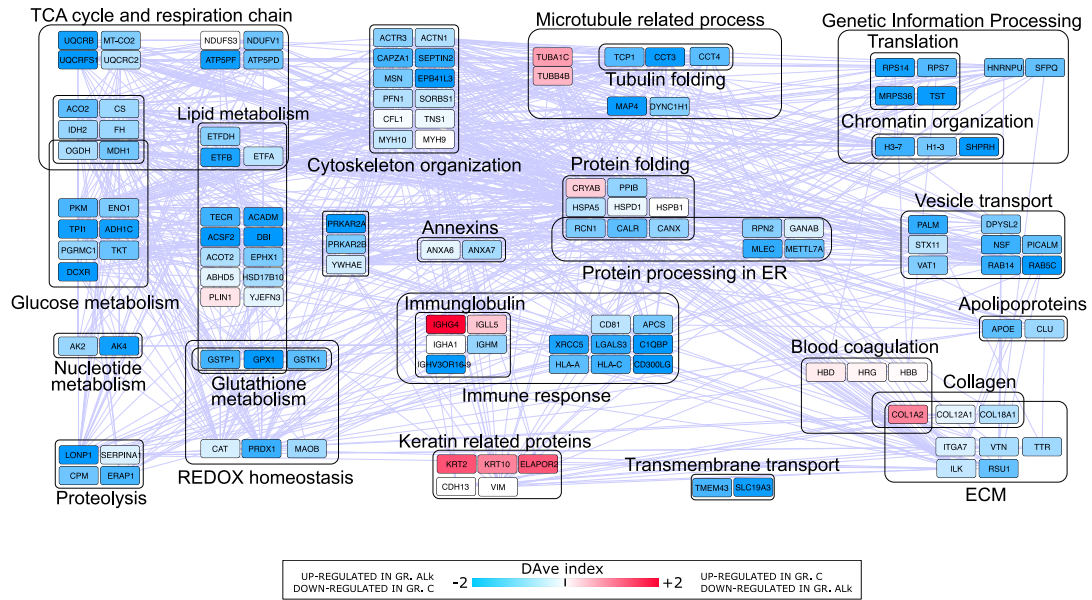

B

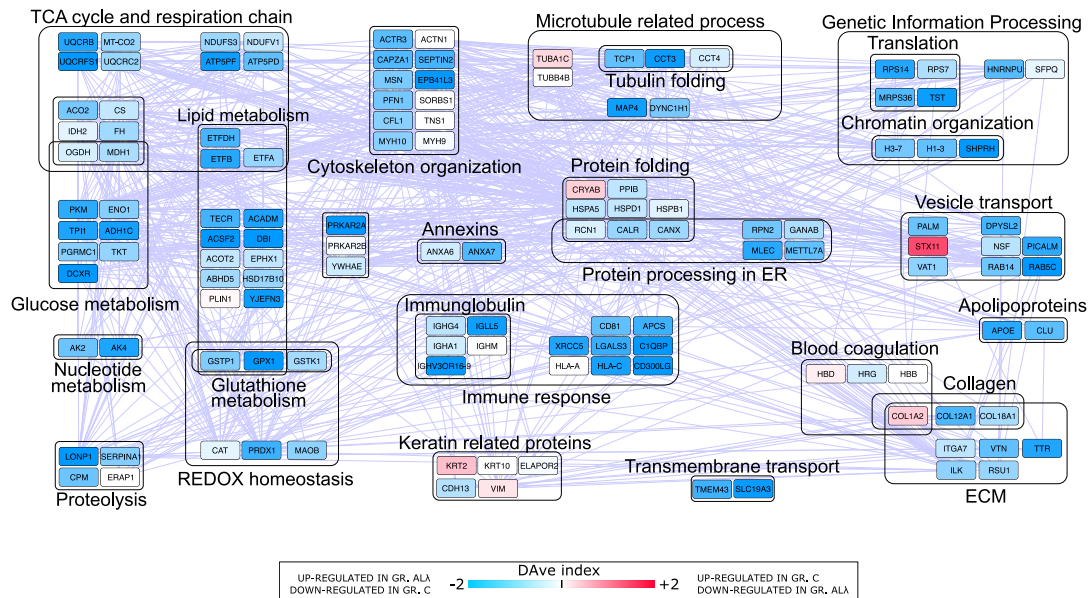

C

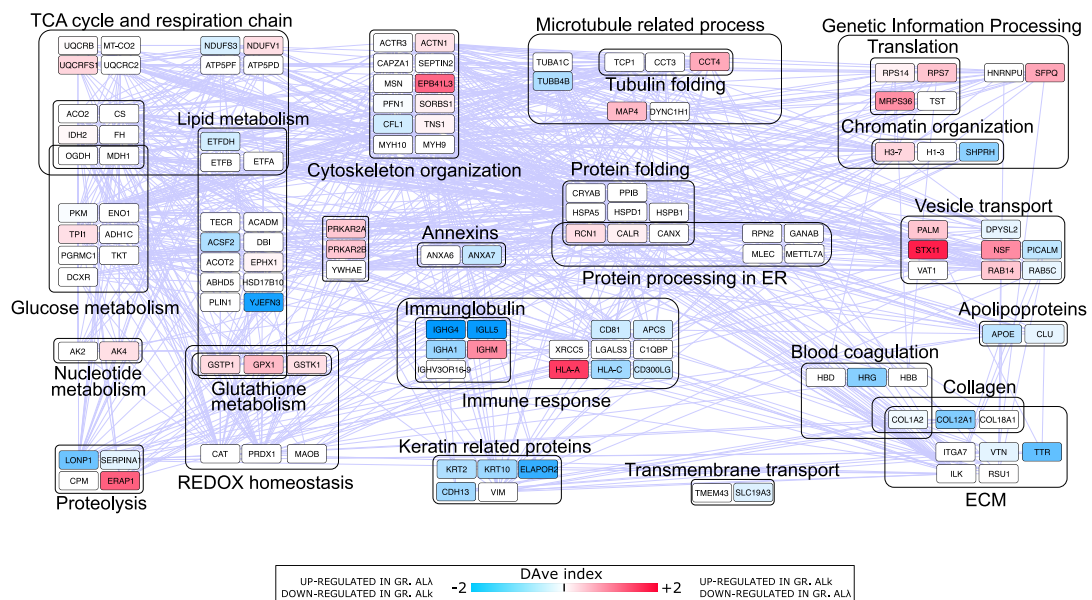

Supplement: Supplementary file 1 [file cells-12-00699-s001.zip › Supplementary files/FigureS2.pdf]

A

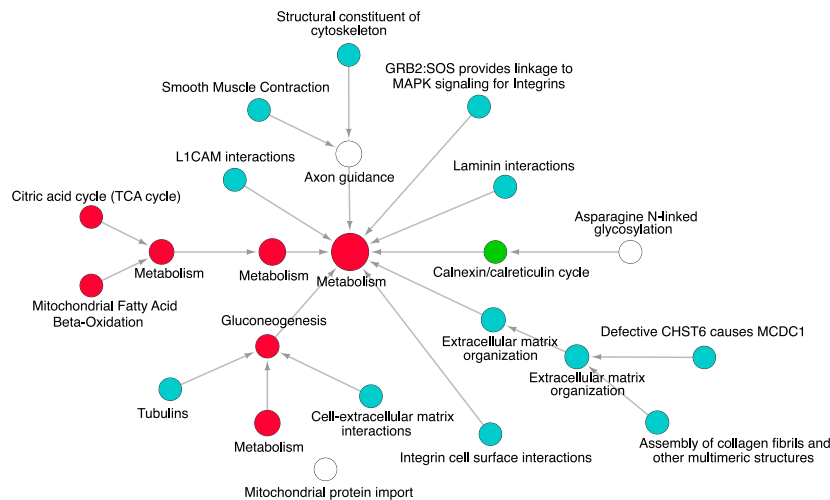

CONTROL

B

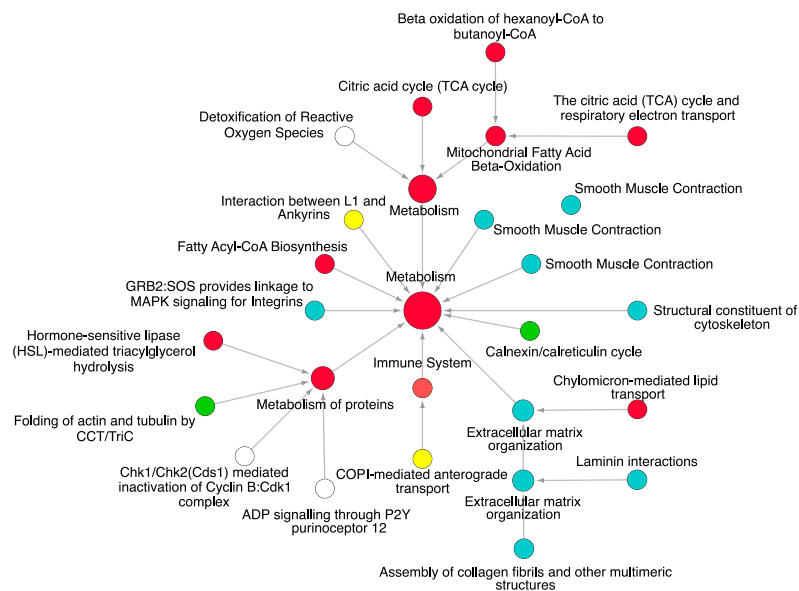

ALK

C

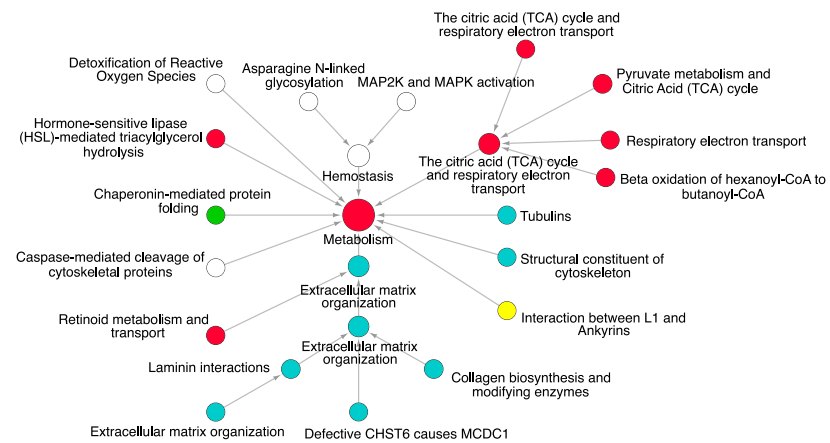

ALA

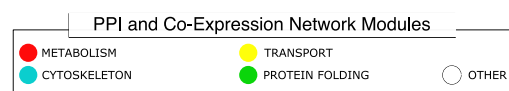

Supplement: Supplementary file 1 [file cells-12-00699-s001.zip › Supplementary files/FigureS3.pdf]

A

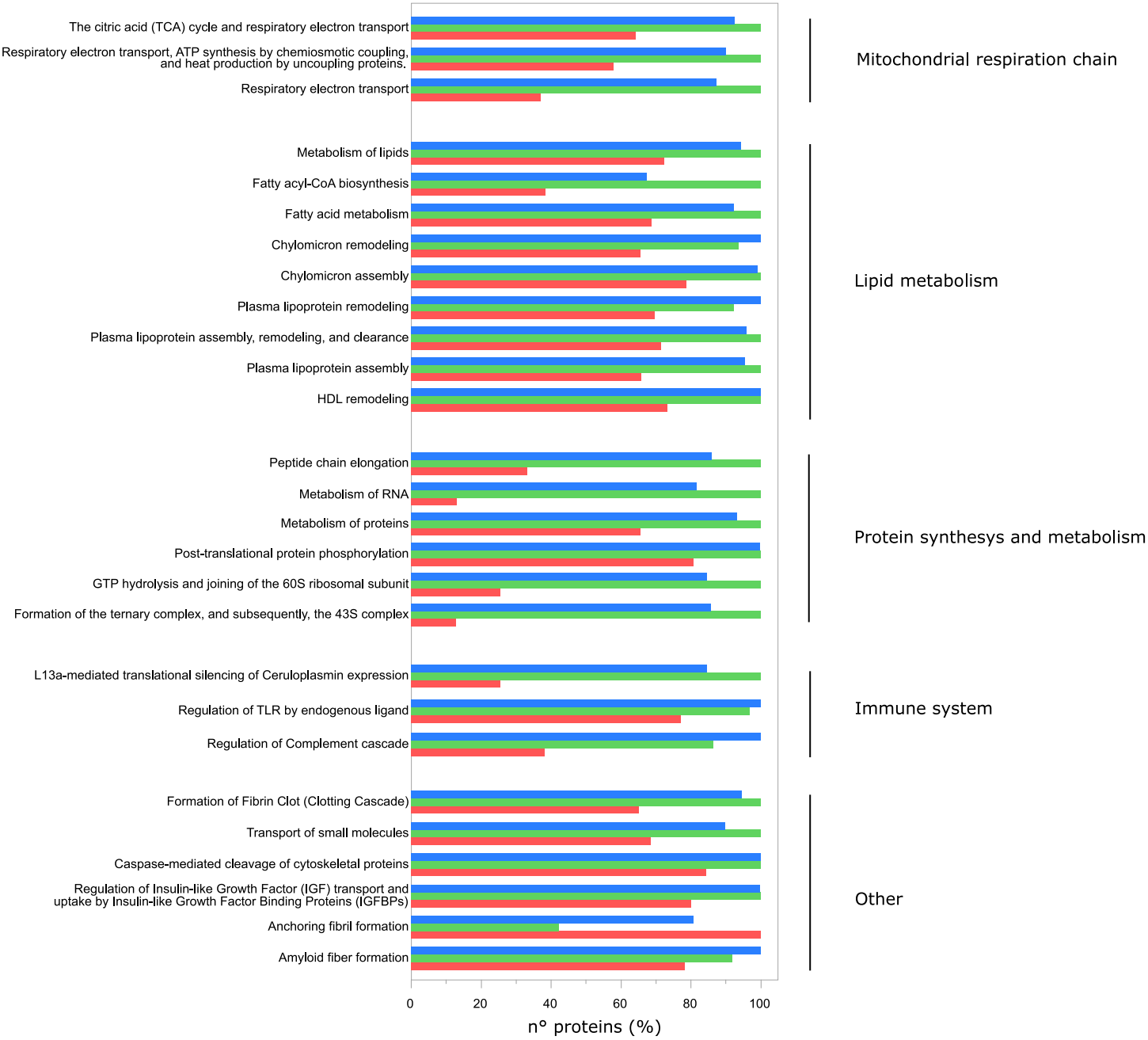

B

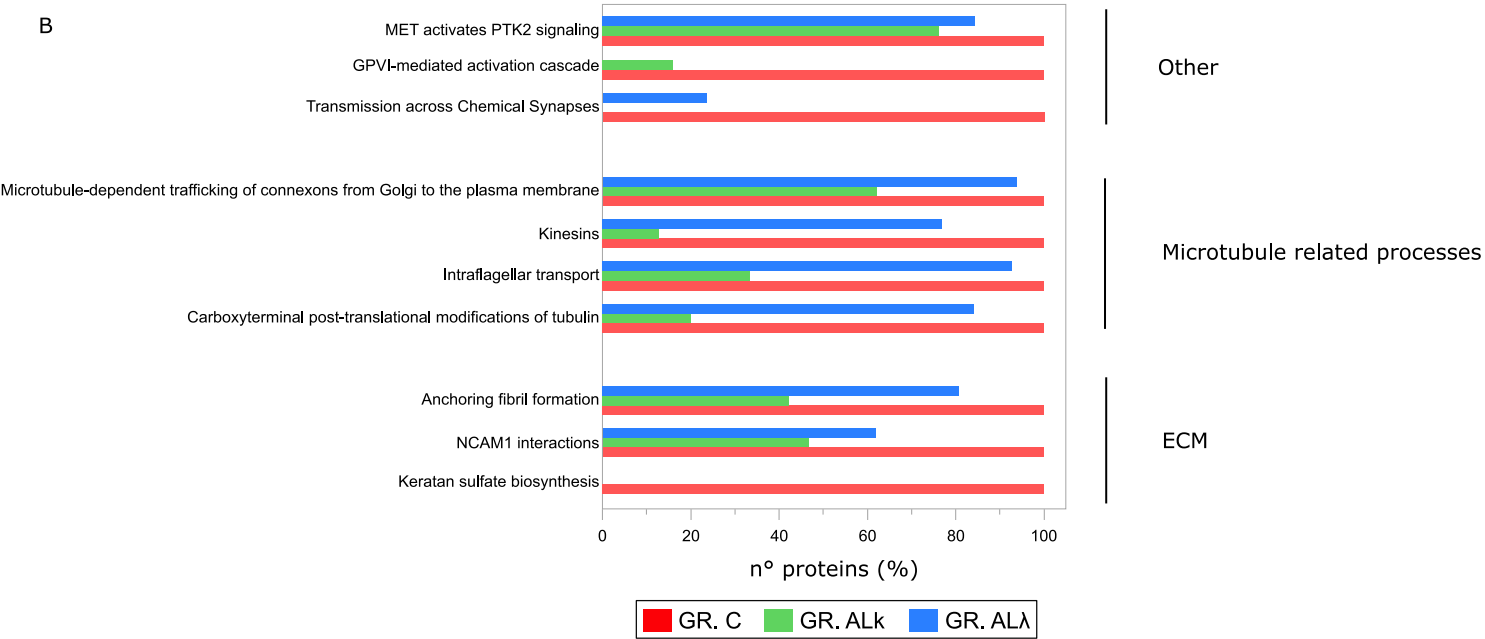

Supplement: Supplementary file 1 [file cells-12-00699-s001.zip › Supplementary files/FigureS1.pdf]

A

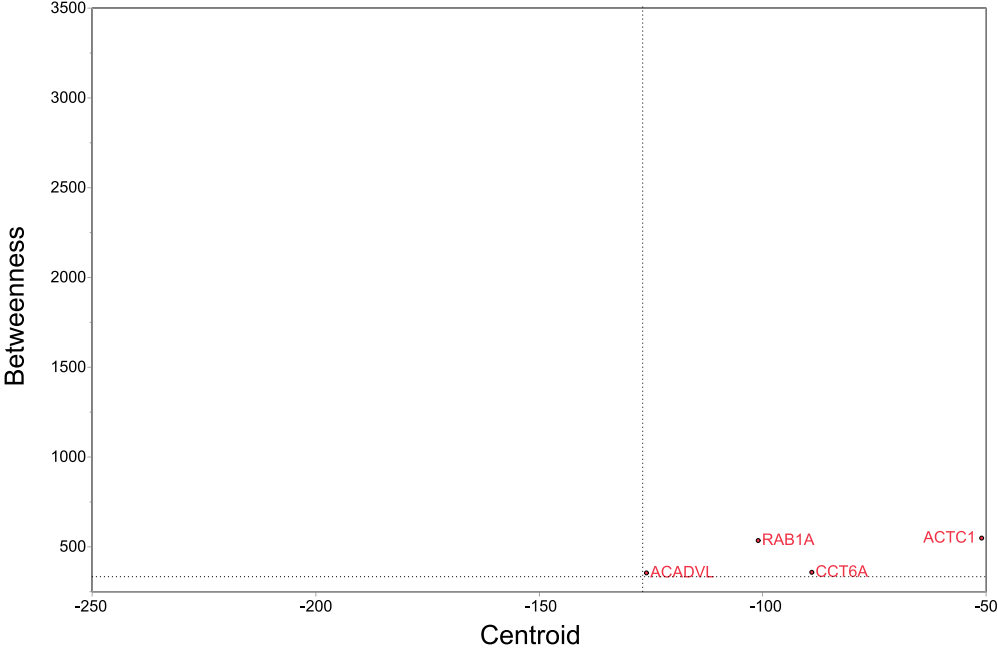

B

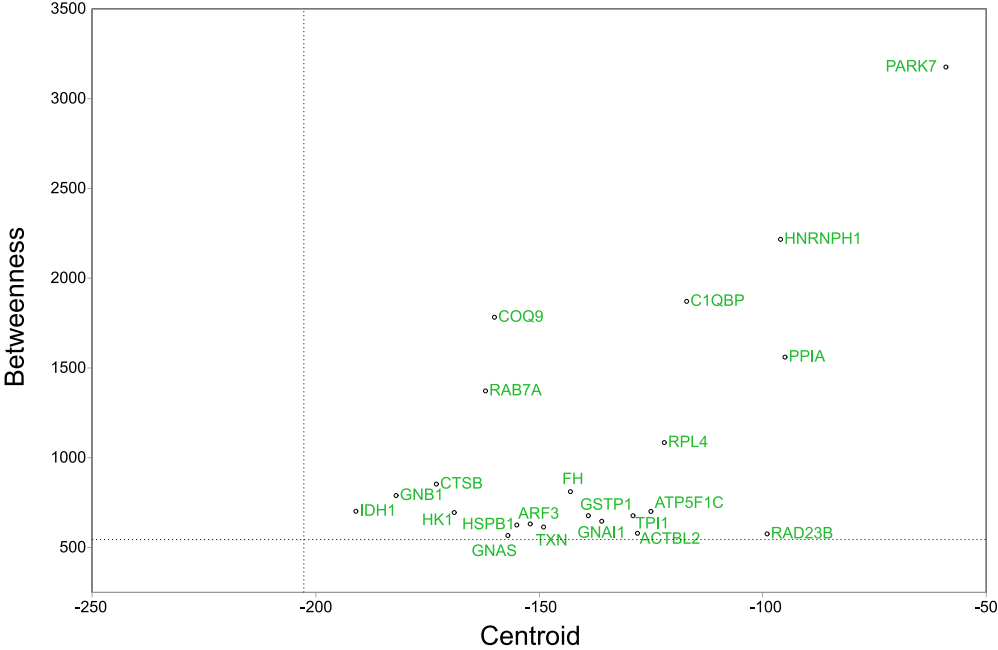

C

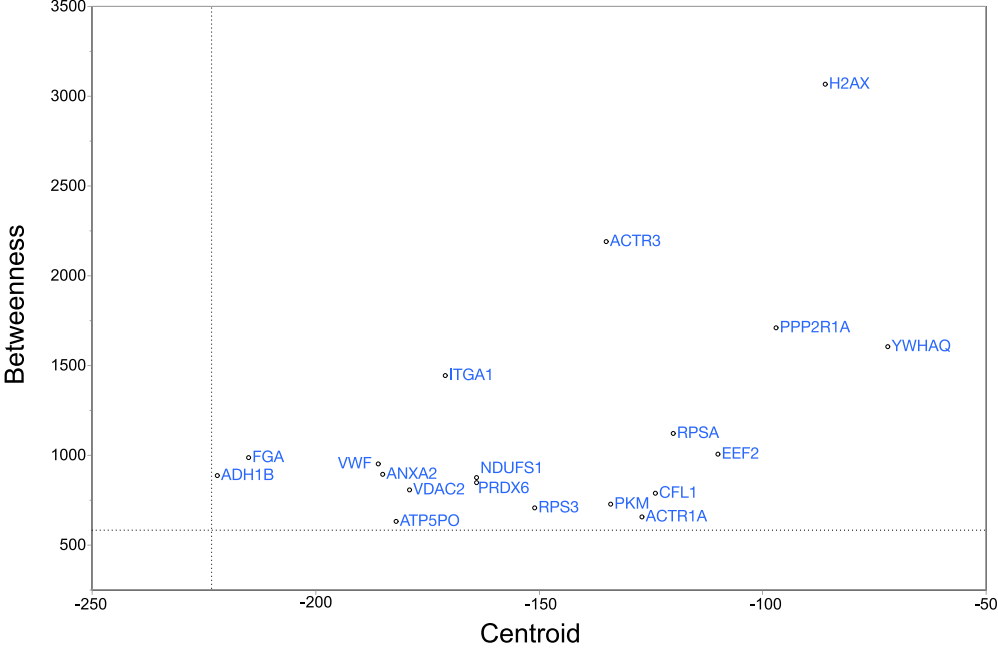

GR, C PPI HUBs - GR, ALk PPI HUBs - GR, ALλ PPI HUBs

Supplement: Supplementary file 1 [file cells-12-00699-s001.zip › Supplementary files/FigureS4.pdf]
